# *Campylobacter jejuni* and *Campylobacter coli* in wild birds on Danish livestock farms

**DOI:** 10.1186/s13028-016-0192-9

**Published:** 2016-02-03

**Authors:** Birthe Hald, Marianne Nielsine Skov, Eva Møller Nielsen, Carsten Rahbek, Jesper Johannes Madsen, Michael Wainø, Mariann Chriél, Steen Nordentoft, Dorte Lau Baggesen, Mogens Madsen

**Affiliations:** 1 Danish Veterinary Laboratory, Department of Poultry, Fish and Fur Animals, 8200 Aarhus N, Denmark; 2 Department of Microbiology, Danish Veterinary Laboratory, 1870 Frederiksberg C, Denmark; 3 Natural History Museum of Denmark, University of Copenhagen, 1350 Copenhagen K, Denmark; 4 The Danish Meatboard, 1609 Copenhagen V, Denmark; 5 Center for Macroecology, Evolution, and Climate, Natural History Museum of Denmark, University of Copenhagen, 2100 Copenhagen Ø, Denmark; 6 National Food Institute, Technical University of Denmark, 2860 Søborg, Denmark; 7 Research Unit for Clinical Microbiology, University of Southern Denmark, 5000 Odense C, Denmark; 8 Department of Microbiology and Infection Control, Statens Serum Institut, 2300 Copenhagen S, Denmark; 9 Imperial College London, Silwood Park Campus, Ascot, Berkshire, SL5 7PY UK; 10 Chr. Hansen, 2970 Hørsholm, Denmark; 11 National Veterinary Institute, Technical University of Denmark, 1870 Frederiksberg C, Denmark; 12 Novo Nordisk, 4400 Kalundborg, Denmark; 13 Dianova Ltd., 8200 Aarhus N, Denmark

**Keywords:** *Campylobacter* spp. epidemiology, *C. jejuni*, *C. coli*, Wild birds, Livestock farms, Ecological guild, Cattle, Pig, Poultry

## Abstract

**Background:**

Reducing the occurrence of campylobacteriosis is a food safety issue of high priority, as in recent years it has been the most commonly reported zoonosis in the EU. Livestock farms are of particular interest, since cattle, swine and poultry are common reservoirs of *Campylobacter* spp. The farm environment provides attractive foraging and breeding habitats for some bird species reported to carry thermophilic *Campylobacter* spp. We investigated the *Campylobacter* spp. carriage rates in 52 wild bird species present on 12 Danish farms, sampled during a winter and a summer season, in order to study the factors influencing the prevalence in wild birds according to their ecological guild. In total, 1607 individual wild bird cloacal swab samples and 386 livestock manure samples were cultured for *Campylobacter* spp. according to the Nordic Committee on Food Analysis method NMKL 119.

**Results:**

The highest *Campylobacter* spp. prevalence was seen in 110 out of 178 thrushes (61.8 %), of which the majority were Common Blackbird (*Turdus merula)*, and in 131 out of 616 sparrows (21.3 %), a guild made up of House Sparrow (*Passer domesticus*) and Eurasian Tree Sparrow (*Passer montanus*). In general, birds feeding on a diet of animal or mixed animal and vegetable origin, foraging on the ground and vegetation in close proximity to livestock stables were more likely to carry *Campylobacter* spp. in both summer (*P* < 0.001) and winter (*P* < 0.001) than birds foraging further away from the farm or in the air. Age, fat score, gender, and migration range were not found to be associated with *Campylobacter* spp. carriage. A correlation was found between the prevalence (%) of *C. jejuni* in wild birds and the proportions (%) of *C. jejuni* in both manure on cattle farms (R^2^ = 0.92) and poultry farms (R^2^ = 0.54), and between the prevalence (%) of *C. coli* in wild birds and the proportions (%) of *C. coli* in manure on pig farms (R^2^ = 0.62).

**Conclusions:**

The ecological guild of wild birds influences the prevalence of *Campylobacter* spp. through the behavioural patterns of the birds. More specifically, wild birds eating food of animal or mixed animal and vegetable origin and foraging on the ground close to livestock were more likely to carry *Campylobacter* spp. than those foraging further away or hunting in the air. These findings suggest that wild birds may play a role in sustaining the epidemiology of *Campylobacter* spp. on farms.

## Background

Human campylobacteriosis has been the most commonly reported zoonosis in the European Union (EU) since 2005, with 214,779 confirmed cases in 2013 according to the European Food Safety Authority (EFSA) [1]. The disease burden was calculated at 35,000 disability-adjusted life years (DALYs) per year and the annual cost in the EU at around €2.4 billion [2]. The global number of DALYs was calculated to be 7,541,000 per year [3]. The cause of campylobacteriosis is *Campylobacter* spp. (primarily *C. jejuni* and *C. coli*)—a Gram-negative, spiral, microaerophilic bacterium and a common commensal inhabitant of the intestinal microflora of food production animals such as cattle, pigs and poultry [4]. It is estimated that 50–80 % of *Campylobacter* spp. strains infecting humans originate from the chicken reservoir, 20–30 % from the cattle reservoir and a small proportion from other reservoirs including wild animals [5]. As a consequence, the entire meat production chain and end products may be contaminated with *C. jejuni* or *C. coli*. In the EU, the pathways to humans are mainly through food, though environmental transmission and direct animal contact are also possible [6]. Therefore, reducing the occurrence of campylobacteriosis in the EU is a food safety issue of high priority, yet one which presents challenges [7].

According to a recent and extensive systematic review of 95 published studies of *Campylobacter* spp. sources around broiler farms [8], several wild animals (including wild birds) are known to be carriers. However, only a small number of the reviewed studies had a primary focus on wild birds living in close proximity to the farms. On a broiler farm in Athens GA, USA, 10 % (of 124) wild birds—mainly House Sparrow (*Passer domesticus*) and Common Starling (*Sturnus vulgaris*)—carried *C. jejuni* [9]. Colles et al. [10] found *C. jejuni* in 50.2 % of droppings from 331 Canada Goose (*Branta canadensis*) and Greylag Goose (*Anser anser*), and in 29.9 % of 954 Common Starling on a free-range broiler farm. Concerning cattle farms, a study in central Iowa, USA sampled 188 wild birds on dairy cattle, sheep and goat farms and found *Campylobacter* spp. in 4.8 % [11].

During the past decade, source attribution studies including multilocus sequence typing (MLST) have been conducted to compare the similarity of *C. jejuni* strains from wild birds with those from chicken and cattle [10–15] and with isolates from human disease [10, 12, 13, 15–17]. The overall conclusion is that the vast majority of *C. jejuni* strains are highly host specific. However, the studies also all identified a small proportion of strains with genotypes overlapping wild birds, farm animals [10–15] and human disease isolates [10, 13, 15–17].

Several studies on *Campylobacter* spp. carriage rates in wild birds in urban areas report a prevalence from 0–90 % [18–24]. Although it would appear that wild birds living in cities (mainly sparrows, pigeons, doves and starlings) have low carriage rates [19, 20, 22], French et al. [16] suggested that wild birds in city parks could contribute to campylobacteriosis in preschool children. The overall highest reported carriage rates have been found in gulls and crows foraging on refuse dumps in urban areas of Norway, Sweden, England, Japan, Spain and USA [18–21, 23–25].

Some of the large discrepancies in wild bird *Campylobacter* spp. prevalence between different studies may be attributed to host taxonomy or differences in the ecological guilds present. Bird ecological guilds are groupings of birds that exploit environmental resources in a similar way [26, 27]. The significance of different ecological guilds on the carriage rates of *Campylobacter* spp. was shown in a study of 1794 birds (the majority of which were migratory), sampled at Ottenby Bird Observatory on the island Oeland, Sweden [28]. The highest prevalence of *Campylobacter* spp. was found among ground-foraging guilds of short-distance migratory birds wintering in Europe.

The aim of our study was to estimate the prevalence of *Campylobacter* spp. in farm related wild bird species. Additionally, to investigate an association between *Campylobacter* spp. contaminated farm environments and wild birds around cattle, pig and poultry farms by performing an analysis of factors associated with *Campylobacter* spp. carriage of the wild birds.

## Methods

### Study design and selection of farms

The study covered four cattle farms, four slaughter pig farms, and four free-range poultry farms in Denmark, together with the wild bird populations living inside production buildings or within a 100 m radius from the farms. The study was conducted during January and February (winter) and during August and September (summer) in 2001. Two farms were sampled per week, and visited every weekday in order to get as many wild bird samples as possible. The cattle and pig farms were initially selected for a project investigating the occurrence of *Salmonella* in wildlife near Danish cattle and pig farms during 2001 and 2002 [29], while the poultry farms were included in this study only. The sampling schemes for *Campylobacter* spp. and *Salmonella* were conducted simultaneously in 2001.

### Sampling

#### Wild birds

Birds were caught and ringed following the EURING system (http://www.euring.org/) by licensed ringers with mist-nets, traps, or by hand, thus ensuring that each bird was only sampled once per sampling event. The birds were released again after sampling. To ensure that a sufficient number of birds were caught during the winter months, several feeding places were established at each herd, using sterilised birdseed. We sampled as many birds as possible, and data on the estimated age, fat score, gender and exact place of capture were noted. Cloacal swab samples were obtained from the wild birds, using slim aluminum cotton swabs (DANSU, Ganløse, Denmark) and placed in Brain Heart Infusion (BHI) transport medium (DIFCO, Sparks, MD, USA) containing 5 % (v/v) calf blood (National Veterinary Institute, Copenhagen, Denmark) and 0.5 % agar (Oxoid Ltd., Basingstoke, Hampshire, UK).

#### Production animals

To detect *Campylobacter* spp. in cattle and pig herds, manure was collected at numerous places in the livestock facilities or among herds in pasture, and mixed into approximately twenty 200 ml containers (Dispatch Container Nunc, Life Technologies, Nærum, Denmark) per herd in each sampling round (i.e. 5–10 manure samples per container equalling 150–180 ml of manure) in order to obtain a representative measure of the within-herd *Campylobacter* spp. status. In order to sample poultry flocks, material from the litter surface was collected on a pair of boot socks whilst walking through the flock’s resting house [30].

### Bacteriological examination and species characterisation

All samples were transported to the laboratory on the sampling day at ambient temperature, refrigerated overnight between 2 and 4 °C, and *Campylobacter* spp. cultivation was initiated the following day. For the number of samples tested, see Table 1.

**Table 1:** *Campylobacter* spp. prevalence and species distribution

Origin of sample	Number of samples	Total number (%) positive	Number of *C. jejuni* (%)	Number of *C. coli* (%)	Number of other *C.* spp. (%)
*Winter*					
Cattle farms					
Wild birds	268	36 (13.4)	22 (8.2)	13 (4.9)	1 (0.4)
Cattle manure	81	36 (44.4)	32 (39.5)	2 (2.5)	2 (2.5)
Pig farms					
Wild birds	288	64 (22.2)	33 (11.5)	27 (9.4)	4 (1.4)
Pig manure	81	72 (88.9)	0 (0.0)	69 (85.2)	3 (3.7)
Poultry farms					
Wild birds	150	16 (10.7)	10 (6.7)	6 (4.0)	0 (0.0)
Poultry manure	8	1 (12.5)	1 (12.5)	0 (0.0)	0 (0.0)
*Summer*					
Cattle farms					
Wild birds	253	38 (15.0)	36 (14.2)	0 (0.0)	2 (0.8)
Cattle manure	83	55 (66.3)	54 (65.1)	0 (0.0)	1 (1.2)
Pig farms					
Wild birds	330	69 (20.9)	68 (20.6)	1 (0.3)	0 (0.0)
Pig manure	83	54 (65.1)	4 (4.8)	50 (60.2)	0 (0.0)
Poultry farms					
Wild birds	318	73 (23.0)	70 (22.0)	1 (0.3)	2 (0.6)
Poultry manure	50	45 (90.0)	41 (82.0)	4 (8.0)	0 (0.0)

The number of samples tested for *Campylobacter* spp., the total number and percentage of positive samples, and the numbers of *C. jejuni, C. coli* and other *Campylobacter* spp. positive samples isolated in wild birds and in livestock manure on each farm type in winter and summer

#### Cloacal swabs

*Campylobacter* spp. were isolated by streaking a swab with the faecal material directly on to modified Charcoal Cefoperazone Deoxycholate Agar (mCCDA) (CM0739, SR0155) (Oxoid) [31], and the plates were incubated under microaerobic conditions (6 % O_2_, 6 % CO_2_, in 88 % N_2_) at 42 °C for 48 h. *Campylobacter* spp.-like colonies were purified on blood agar and identified to species level using standard procedures including tests for hippurate and indoxyl acetate hydrolysis, catalase production and susceptibility to cephalotin and nalidixic acid according to NMKL 119 [32]. *Campylobacter* spp. isolates were identified as *C. jejuni, C. coli,* *C. lari*, *C.* *upsaliensis,* *C. hyointestinalis* or *Campylobacter* spp.

#### Manure

The manure was diluted to 1 g per 9 ml of buffered peptone water (CM1049, Oxoid), and 10 µl of the suspended material was streaked on mCCDA and incubated as described above.

#### Boot socks

Each pair of boot socks was placed in a stomacher bag, and after being diluted in 1:10 w/w in buffered peptone water (CM1049, Oxoid), faeces were released by gentle manipulation and 10 µl of the suspension was spread on mCCDA and incubated as described above.

### Data analysis

The dependent variable was defined as a positive isolation of *Campylobacter* spp. from a wild bird. Descriptive statistics were performed using bivariate analysis [33] on *Campylobacter* spp. positive samples from wild birds. The association between independent variables was assessed using the Chi square test with a statistical significance threshold of *P* < 0.05. The evaluation of a possible association between *Campylobacter* spp. positive samples in the wild birds and in the herd was carried out separately for the two seasons (winter and summer).

Six potential factors associated with *Campylobacter* spp. carriage were included: (1) age (old, young); (2) herd type (cattle, pig, poultry); (3) proximity (in stable, around stable); (4) ecological guild with ≥10 samples (i.e. aerial insectivorous, foliage-gleaners, insectivorous seedeaters, open-land insectivorous, tit-like birds, sparrows, passerine seedeaters, terrestrial and low fly-catching feeders and thrushes); (5) fat score (0–8) [34], and (6) gender (male, female, not determined).

Based on the characteristic behaviour patterns of each ecological guild, the following five factors were selected: (1) feed (animal, mix, vegetable); (2) forage area (aerial, ground, vegetation); (3) proximity to stables (in stable, around stable); (4) contact with slurry (no, yes), and (5) migration range (long, medium, short, partial, none). This analysis included only the summer sampling, as more guilds were present, and the birds exhibited a wider range of behavioural patterns during the summer season than in winter.

Multivariate analyses [33] were carried out in all sampled wild birds organised in an ecological guild structure based on Gotellia et al. [27], using SAS Enterprise guide ver. 3.0.2. The logistic regression analyses were carried out using SAS PROC GENMOD. The modelling procedure assumed a binomial distribution and used logit as the link function. Goodness of fit was assessed by likelihood ratio statistics. The model was adjusted for overdispersion using the PSCALE option. In the analysis, non-significant variables were removed using stepwise backwards elimination. Statistical significance of the covariates was assessed using the likelihood ratio test based on *P*  ≤  0.05. The odds ratio (OR) and the 95 % confidence interval were reported for statistically significant variables.

In order to evaluate the impact of different herd types and season on the *C. jejuni* and *C. coli* carriage rates, sparrows (n = 616) were selected for the analysis, since this guild of non-migratory wild birds was the only one to be caught in a sufficient number on all farms during both winter and summer sampling. Correlation coefficients (R^2^) were calculated between the prevalence (%) of *C. jejuni* and *C. coli* in sparrows and the proportions (%) *C. jejuni* and *C. coli* in manure from each of the three herd types.

## Results

### *Campylobacter* spp. prevalence in sampled wild birds

In total, 1607 wild birds were sampled. The overall *Campylobacter* spp. carriage rate was significantly lower in winter (15.9 %, 112 positive samples out of a total of 706) than in summer (20.0 %, 180 positive samples out of a total of 901; OR = 1.32, 1.02–1.71, *P* = 0.03). For the species of *Campylobacter* spp. detected in each farm type, and the carriage rate among wild birds in winter and summer, see Table 1. For the prevalence of *Campylobacter* spp. in each bird species, see Table 2 and grouped in ecological guilds , see Table 3.

**Table 2:** The prevalence of *Campylobacter* spp. in wild birds and the allocation of bird species to ecological guild

Ecological guilds	Species	Common name	Number tested W/S	Number positive W/S	% *Campylobacter* positive W/S
Aerial insectivorous	*Delichon urbicum*	Common house martin	0/83	0/0	0.0/0.0
	*Delichon urbicum* (brood)	Common house martin, brood	0/2	0/0	0.0/0.0
	*Hirundu rustica*	Barn swallow	0/128	0/10	0.0/7.8
	*Hirundu rustica* (brood)	Barn swallow, brood	0/21	0/4	0.0/19.0
Bud-browser and seedeaters	*Pyrrhula pyrrhula*	Eurasian bullfinch	1/5	0/0	0.0/0.0
Columbids	*Columba livia domesticus*	Feral pigeon	3/3	1/0	33.3/0.0
	*Columba palumbus*	Common wood pigeon	0/1	0/0	0.0/0.0
	*Streptopelia decaocto*	Eurasian collared dove	2/3	0/0	0.0/0.0
Flycatcher	*Muscicapa striata*	Spotted flycatcher	0/2	0/1	0.0/50.0
Foliage-gleaners	*Fringilla coelebs*	Common chaffinch	26/2	0/0	0.0/0.0
	*Hippolais icterina*	Icterine warbler	0/1	0/0	0.0/0.0
	*Phylloscopus collybita*	Common chiffchaff	0/12	0/0	0.0/0.0
	*Phylloscopus trochilus*	Willow warbler	0/18	0/3	0.0/16.7
	*Sylvia atricapilla*	Eurasian blackcap	0/9	0/2	0.0/22.2
	*Sylvia borin*	Garden warbler	0/9	0/0	0.0/0.0
	*Sylvia communis*	Common whitethroat	0/44	0/5	0.0/11.4
	*Sylvia curruca*	Lesser whitethroat	0/9	0/3	0.0/33.3
Gallinaceous birds	*Phasianus colchicus*	Common pheasant	1/0	0/0	0.0/0.0
Gulls	*Larus canus*	Mew gull	2/0	0/0	0.0/0.0
Insectivorous seedeaters	*Emberiza citrinella*	Yellowhammer	2/19	0/3	0.0/15.8
	*Emberiza calandra*	Corn bunting	0/3	0/1	0.0/33.3
Marshwarblers	*Acrocephalus palustris*	Marsh warbler	0/5	0/0	0.0/0.0
	*Acrocephalus scirpaceus*	Eurasian reed warbler	0/1	0/0	0.0/0.0
Omnivorous corvidae	*Corvus frugilegus*	Rook	2/0	0/0	0.0/0.0
Open-land insectivorous	*Alauda arvensis*	Eurasian skylark	0/1	0/0	0.0/0.0
	*Anthus trivialis*	Tree pipit	0/1	0/0	0.0/0.0
	*Motacilla alba*	White wagtail	0/7	0/4	0.0/57.1
	*Motacilla alba* (brood)	White wagtail, brood	0/1	0/1	0.0/100.0
Passerine seedeaters	*Carduelis cannabina*	Common linnet	0/5	0/0	0.0/0.0
	*Carduelis carduelis*	European goldfinch	0/3	0/0	0.0/0.0
	*Carduelis chloris*	European greenfinch	70/20	0/1	0.0/5.0
	*Carduelis flammea*	Common redpoll	3/0	0/0	0.0/0.0
Scolopacids	*Tringa ochropus*	Green sandpiper	0/1	0/0	0.0/0.0
Sparrows	*Passer domesticus*	House sparrow	214/152	38/51	17.8/33.6
	*Passer montanus*	Eurasian tree sparrow	81/169	1/41	1.2/24.3
Stream specialist	*Motacilla cinerea*	Grey wagtail	0/1	0/0	0.0/0.0
Terrestrial and low fly-catching feeders	*Erithacus rubecula*	European robin	25/4	0/0	0.0/0.0
	*Luscinia luscinia*	Thrush nightingale	0/1	0/0	0.0/0.0
	*Oenanthe oenanthe*	Northern wheatear	0/1	0/0	0.0/0.0
	*Phoenicurus phoenicurus*	Common redstart	0/3	0/0	0.0/0.0
	*Prunella modularis*	Dunnock	9/9	4/2	44.4/22.2
	*Saxicola rubetra*	Whinchat	0/3	0/0	0.0/0.0
	*Troglodytes troglodytes*	Eurasian wren	16/18	0/0	0.0/0.0
Thrushes	*Turdus merula*	Common blackbird	119/55	63/44	52.9/80.0
	*Turdus pilaris*	Fieldfare	3/0	3/0	100/0.0
	*Turdus viscivorus*	Mistle thrush	1/0	0/0	0.0/0.0
Tit-like birds	*Certhia brachydactyla*	Short-toed treecreeper	1/0	0/0	0.0/0.0
	*Certhia familiaris*	Eurasian treecreeper	0/1	0/0	0.0/0.0
	*Cyanistes caeruleus*	Eurasian blue tit	30/15	0/0	0.0/0.0
	*Lophophanes cristatus*	European crested tit	1/0	0/0	0.0/0.0
	*Parus major*	Great tit	86/43	2/1	2.3/2.3
	*Poecile palustris*	Marsh tit	5/3	0/0	0.0/0.0
	*Regulus regulus*	Goldcrest	1/0	0/0	0.0/0.0
	*Sitta europaea*	Eurasian nuthatch	1/0	0/0	0.0/0.0
No guild	*Bombycilla garrulus*	Bohemian waxwing	1/0	0/0	0.0/0.0
	*Sturnus vulgaris*	Common starling	0/4	0/3	0.0/75.0

The species and number of birds tested for *Campylobacter* spp., and the prevalence in each bird species in winter (W) and summer (S)

**Table 3:** *Campylobacter* spp. prevalence in ecological guilds

Guild	Winter			Summer		
	Number of samples	Prevalence (%)	OR (95 % CI)	Number of samples	Prevalence (%)	OR (95 % CI)
Aerial insectivorous	–	–	–	234	6.0	0.2 (0.1–0.3)
Foliage-gleaners	26	0.0	NA^a^	104	12.5	0.4 (0.2–0.7)
Insectivorous seedeaters	–	–	–	22	18.2	0.6 (0.2–1,7)
Open-land insectivorous	–	–	–	10	50.0	2.5 (0.7–8.8)
Passerine seedeaters	73	0.0	NA	28	3.6	0.1 (0.01–0.7)
Sparrows	295	13.2	1.0 (reference)	321	28.7	1.0 (reference)
Terrestrial and low fly catching feeders	50	8.0	0.6 (0.2–1.7)	39	5.1	0.1 (0.03–0.6)
Thrushes	123	53.7	7.6 (4.6–12.4)	55	80.0	9.9 (4.9–20.1)
Tit-like birds	125	1.6	0.1 (0.03–0.5)	62	1.6	0.04 (0.01–0.3)
Total	692			875		

The odds-ratios (OR) and 95 % confidence interval (95 % CI) from the multivariate analysis of *Campylobacter* spp. prevalence in ecological guilds with ≥10 birds sampled in winter and summer, with sparrows used as a reference

^a^Not applicable due to zero positive samples

The *Campylobacter* spp. carriage rates varied considerably between ecological guilds. The highest prevalence was found within two guilds: thrushes with 61.8 % (110/178) positive samples and sparrows with 21.3 % (131/616) positive samples (Table 2). Combined, these guilds were responsible for 82.5 % (241 out of 292) of the positive wild bird samples. The main bird species of these two guilds were the Common Blackbird (*Turdus merula*; n = 174), House Sparrow (n = 366) and Eurasian Tree Sparrow (*Passer montanus*; n = 250). They were also the most frequently sampled wild birds on the farms. Other birds that were frequently present were the Barn Swallow (*Hirundu rustica*; n = 128), Great Tit (*Parus major*; n = 129), European Greenfinch (*Carduelis chloris*; n = 90) and Common House Martin (*Delichon urbica*; n = 83), all of which had a low *Campylobacter* spp. prevalence (Table 2).

### Factors associated with *Campylobacter* spp. carriage in wild birds

Analysis of the six selected risk factors for *Campylobacter* spp. carriage in wild birds (age, herd type, proximity, ecological guild, fat score and gender) revealed that the ecological guild was significantly associated with *Campylobacter* spp. carriage during both winter and summer (Table 3). Thrushes and open-land insectivorous birds were more likely to carry *Campylobacter* spp. than sparrows (used as a reference guild), whereas all other guilds had lower odds than sparrows. In general, herd type, fat score, gender and age were not significantly associated with *Campylobacter* spp. prevalence in wild birds (all sampled birds). Proximity was significant in summer (see proximity to stables in Table 4) but not in winter (data not shown).

**Table 4:** Factors associated with *Campylobacter* spp. carriage and specific bird behaviour during summer

Factor	Odds Ratio (95 % CI)
Feed	
Animal origin	8.0 (4.3–15.0)
Mixed animal and vegetable origin	22.6 (7.4–68.6)
Vegetable origin	1.0 (reference)
Forage area	
In the air	0.03 (0.0–0.1)
On the ground	1.03 (0.4–2.8)
In the vegetation	1.0 (reference)
Proximity to stables	
In or at stables	42.72 (14.2–128.5)
Around stables	1.0 (reference)

#### Patterns of behaviour in summer

Concerning the impact of particular patterns of behaviour in summer (i.e. feed, forage area, proximity to stables, contact with slurry and migration range), there was significantly increased odds for *Campylobacter* spp. carriage in birds eating food of animal or mixed animal and vegetable origin foraging on the ground and in vegetation close to the production buildings (Table 4). No association was found between *Campylobacter* spp. carriage and contact with slurry or migration range (data not shown).

### Herd type and *Campylobacter* species distribution

*C. jejuni* was the most commonly isolated *Campylobacter* species in wild birds on all farm types, comprising 78.3 % (58 out of 74) of wild bird isolates on cattle farms, 75.9 % (101 out of 133) on pig farms and 89.9 % (80 out of 89) on poultry farms (Table 1). The remaining isolates were almost entirely *C. coli,* of which 46 out of 48 isolates were found at the winter sampling.

**Fig. 1:**
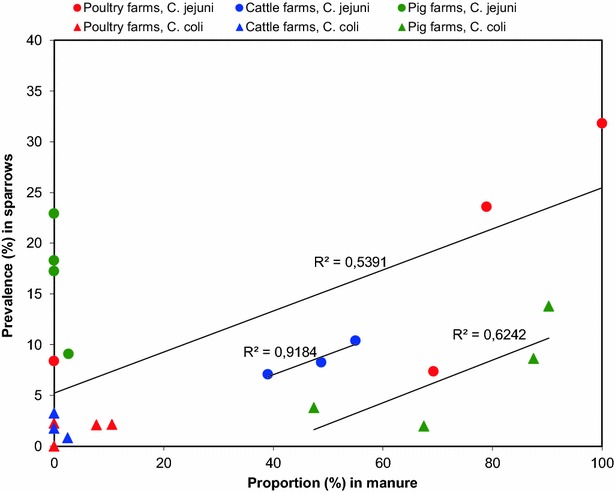
Correlation between the prevalence (%) of *Campylobacter jejuni* and *C. coli* in sparrows and the proportions (%) of *C. jejuni* and *C. coli* in manure from cattle, pig and poultry herds. The prevalence, proportion and correlation coefficients (R^2^) on the regression lines are shown in *red* (poultry farms), *blue* (cattle farms) and *green* (pig farms) *circles* (*C. jejuni*) and *triangles* (*C. coli*)

Looking at the proportions of *Campylobacter* species in herd manure and the prevalence in wild birds at each of the 12 individual farms revealed a strong correlation between the prevalence of *C. jejuni* in both wild birds and the proportions in manure on cattle farms (R^2^ = 0.92), and a moderate correlation on poultry farms (R^2^ = 0.54). Likewise, a moderate correlation was found between *C. coli* in both wild birds and in pig manure (R^2^ = 0.62; Fig. 1). In contrast, no correlation was seen between *C. coli* in wild birds and in manure on cattle and poultry farms, or between *C. jejuni* in wild birds and in manure in pig herds (Fig. 1).

## Discussion

A seasonal peak in the prevalence of *Campylobacter* spp. in wild birds was observed in summer. This was also found in a study of farm related Common Starling in the UK [12], and a study of Black-headed Gull (*Larus ridibundus*) in Sweden [23]. The underlying causes of seasonality in the epidemiology of *Campylobacter* spp. are not fully understood. However, seasonality is also a recognised factor in the pattern of *Campylobacter* spp. infections in poultry [2], and in the occurrence of human campylobacteriosis [1]. The vast majority (82.5 %) of *Campylobacter* spp. in wild birds in our study was isolated from thrushes and sparrows (Tables 2, 3), representing some of the most common wild bird species in Denmark (i.e. Common Blackbird, House Sparrow and Eurasian Tree Sparrow).

The *Campylobacter* spp. carriage rates of the farm-related wild birds were found to be closely associated with the ecological guild (Table 3). Studies from Sweden [28] and Italy [35] have reported results for ecological guilds sampled at bird stations. The Swedish study found the highest *Campylobacter* spp. prevalence in wagtails, Common Starling and thrushes [28], in agreement with the results presented here. Common bird species such as the European Greenfinch, European Robin (*Erithacus rubecula*), Great Tit and Common Chaffinch (*Fringilla coelebs*) showed low *Campylobacter* spp. prevalence in both the Swedish study and the present study (Table 2). Our analysis identified feeding habit, forage area and proximity to stables as factors significantly associated with the carriage of *Campylobacter* spp. in wild birds (Table 4). This is in line with the results of the Italian study [35], where feeding habit was considered an important factor, and carnivorous birds foraging on the ground showed the highest prevalence of *Campylobacter* spp. A Japanese study [20] examined the correlation between the crop and actual stomach content and the prevalence of *C. jejuni,* and found a negative correlation between vegetable stomach content and *C. jejuni* colonisation. Several other studies have reported that omnivorous birds such as crows and gulls foraging close to areas with human garbage and sewage have a particular risk of high carriage rates [19, 20, 24, 25].

We found a correlation between the prevalence of *C. jejuni* in wild birds and proportions in both manure on cattle and poultry farms, and between *C. coli* in wild birds and pig manure (Fig. 1). However, this correlation can only account for part of the *Campylobacter* spp. epidemiology on the farms, since some of the *C. jejuni* and *C. coli* detected in the wild birds (i.e. the *C. jejuni* in birds on pig farms and the *C. coli* in birds on the cattle farms) could not be explained by the correlation to farm manure (Fig. 1, Table 1). It is likely that bird-to-bird transmission, or sources not included in this study were responsible for the observed *Campylobacter* spp. It is also possible that the farm animals and the wild birds both acquired *Campylobacter* spp. from the same sources, but became colonised by different species adapted to their specific gut environments. An interesting aspect for further research would be to investigate why the isolation rate of *C. coli* in the wild birds during the summer sampling was so low on all farms, and why the proportion of *C. coli* in the pig manure was also lower in summer (60.2 %) than in winter (85.2 %; Table 1).

Our study showed that in summer, sparrows caught at poultry or pig farms were more likely to carry *Campylobacter* spp. than sparrows caught at cattle farms. The reason for this remains speculative, though the majority of cows were at pasture during the summer months, thus potentially resulting in minimal contact with the sparrows close to the farm buildings. Further investigation should be performed in order to evaluate this.

We anticipated that wild birds and livestock occupying very close living space might share strains locally and that this might be a key point to understand the epidemiology of *Campylobacter* spp. in wild birds on livestock farms. We realise however, that our study suffers from an inferior resolution depth, as we summarised our results at the *Campylobacter* species level and not the genotype level. We may therefore have emphasised farm factors over strain factors, which were not measured. More recent studies using MLST have shown a large degree of host specificity [12, 17, 36, 37] and minimal overlap in MLST profiles of *Campylobacter* spp. from wild birds and from poultry, cattle and humans. There was a greater similarity between the level of *C. jejuni* found in Common Starling in Sweden and Common Starling in the UK, than there was between *C. jejuni* from Swedish Common Starling and their Swedish environment [37]. This segregation between the *Campylobacter* spp. strains in wild birds and the livestock reservoir is supported by a host attribution study [38] investigating the host association in seven housekeeping loci in 2732 published *C. jejuni* isolates from a number of sources including chicken, farm ruminants, and wild birds (passerine birds, ducks and geese). The main finding was that phylogenetically distinct *C. jejuni* lineages were associated with distinct wild birds, whereas in the farm environment, phylogenetically distant farm animals shared several *C. jejuni* lineages. Likewise, a possible adaptation of certain clonal complexes to flocks of barnacle geese in Finland has been found in a recent study [39]. Some studies note that wild birds may have a minor role in transmitting pathogenic *C. jejuni* strains to cattle [11, 13, 15] and to humans [10, 13, 15, 16, 39], whereas others found no evidence of transmission [12]. A recent study [40] found wild bird *C. jejuni* strains to be a consistent source of human disease in the UK, suggesting the existence of some more obscure epidemiological pathways between the wild bird reservoir and humans. From 2003 to 2013, the burden of campylobacteriosis cases attributed to wild birds was estimated at 10,000 per year in the UK. Therefore, it appears that the development of methods to control the transmission of *Campylobacter* spp. between livestock, humans, and wild birds requires better elucidation and understanding of the dynamics of transmission.

## Conclusions

Based on the findings in this study, we conclude that the carriage of *C. jejuni* and *C. coli* in wild birds on livestock farms is correlated to the proximity to stables, feeding habits and forage areas on the ground and in vegetation. Birds with forage areas further away from livestock buildings or in the air, carried less *Campylobacter* spp. These findings suggest that wild birds may play a role in sustaining the epidemiology of *Campylobacter* spp. on farms, although this study is not able to elucidate the direction of the transmission, and further studies including genotyping are required.
